# Pattern separation performance is decreased in patients with early multiple sclerosis

**DOI:** 10.1002/brb3.739

**Published:** 2017-06-21

**Authors:** Vincent Planche, Aurélie Ruet, Julie Charré‐Morin, Mathilde Deloire, Bruno Brochet, Thomas Tourdias

**Affiliations:** ^1^ University of Bordeaux Bordeaux France; ^2^ Neurocentre Magendie Inserm U1215 Bordeaux France; ^3^ CHU de Clermont‐Ferrand Clermont‐Ferrand France; ^4^ CHU de Bordeaux Bordeaux France

**Keywords:** dentate gyrus, episodic memory, hippocampus, multiple sclerosis, pattern separation

## Abstract

**Background:**

Hippocampal‐dependent memory impairment is frequent and occurs early during the course of multiple sclerosis (MS). While mechanisms responsible for episodic memory dysfunction in patients with MS remain largely unknown, dentate gyrus structure has been suggested as particularly vulnerable at the early stage of the disease. If true, we hypothesized that the pattern separation component of episodic memory (a function known to be critically dependent to dentate gyrus function) would be impaired in patients with early MS (PweMS).

**Methods:**

Thirty eight participants (19 PweMS and 19 healthy controls matched on age, gender and education level) were tested with a behavioral pattern separation task and also for information processing speed and visuospatial episodic memory.

**Results:**

We report a significant decrease in pattern separation performance in PweMS compared to healthy controls (27.07 vs. 40.01, *p* = .030 after Holm–Bonferroni correction, *d* = 1.02) together with a significantly higher pattern completion rate (56.11 vs. 40.95, *p* = .004 after Holm–Bonferroni correction, *d* = 1.07) while no difference was found among groups for information processing speed and “global” visuospatial episodic memory regarding learning, long‐term recall or recognition.

**Conclusion:**

Our results suggest that behavioral pattern separation task can detect subtle memory decline in patients with MS and argue for early dentate gyrus dysfunction during the course of the disease.

## 1 INTRODUCTION

Memory impairment is frequent in multiple sclerosis (MS) (Chiaravalloti & DeLuca, 2008; Rocca, et al., 2015) and can occur very early during the course of the disease (Feuillet et al., 2007). Several prior studies support that hippocampal dysfunction primarily mediates memory impairment in MS, both in the human disease (Sicotte et al., 2008; Dutta et al., 2011; Hulst et al., 2012; Planche et al., 2016) and in animal models such as experimental autoimmune encephalomyelitis (EAE) (Di Filippo et al., 2016; Nisticò et al., 2013; Planche et al., 2017). However, the precise neuropsychological and anatomical mechanisms leading to loss of episodic memory performance still remain poorly understood. Especially, hippocampal subfields might be differentially vulnerable to early inflammatory processes. Regarding this issue, our group has recently provided compelling evidence for an early dentate gyrus vulnerability prior to any other subfields in EAE‐mice (Planche et al., 2017). We have indeed demonstrated that dentate gyrus granule cells were the most vulnerable hippocampal neurons at the early stage of the disease and that this selective neurodegenerative process was sufficient to cause memory impairment. Translation of these findings to patients with MS is highly challenging due to difficulty in isolating and quantifying precisely the alterations within the dentate gyrus in human brain. Therefore, MRI studies in patients with MS have reported controversial results, possibly due to different postprocessing techniques, and have highlighted a differential vulnerability either of CA1 (Longoni et al., 2015; Sicotte et al., 2008) or of CA3/CA4/dentate gyrus (Gold et al., 2010; Rocca, et al., 2015). Therefore, whether the concept of an early selective vulnerability of the dentate gyrus is true in patients with early MS remains to be demonstrated.

A fundamental physiological role of hippocampus is to allow the formation of new memories as individual representations. The anatomical and functional “front door” of hippocampus is the dentate gyrus where the perforant path/dentate granule cells system eliminates repetitive information to avoid interference between long‐term memory and new sensory inputs (Rolls, 2013). This computational view of dentate gyrus function is known as pattern separation, which is the process of establishing independent and nonoverlapping new memories (Yassa & Stark, 2011). The critical role of dentate gyrus in pattern separation has been well established with behavioral, specific subfields lesions, gene knock‐out and electrophysiological experiments in rodents (Goodrich‐Hunsaker, Hunsaker, & Kesner, 2008; Leutgeb, Leutgeb, Moser, & Moser, 2007; McHugh et al., 2007; Neunuebel & Knierim, 2014) but also with functional MRI in humans (Bakker, Kirwan, Miller, & Stark, 2008; Berron et al., 2016; Lacy, Yassa, Stark, Muftuler, & Stark, 2011) where representation of similar events was always less overlapping in dentate gyrus than in the other hippocampal subfields (Berron et al., 2016). The complementary process to pattern separation is pattern completion, defined as the ability to complete a whole memory when just a partial cue for retrieval is presented. Pattern completion is a function classically attributed to the CA3 subfield of hippocampus. Indeed, CA3 (and its projections to CA1) integrates the new pattern of information with previous memories, even when partial or degraded versions of the original inputs are presented (Rolls, 2013).

The investigation of people with early MS (PweMS) provides the opportunity to understand the mechanisms underlying episodic memory deficits before the occurrence of other cognitive impairments and before the emergence of global brain damages. Then, comprehensive neuropsychological evaluations of PweMS have already pointed deficits in acquisition/encoding of new memories more than impairments in storage, retrieval or recognition (DeLuca, Gaudino, Diamond, Christodoulou, & Engel, 1998; Müller et al., 2013; Olivares et al., 2005). In this context, if dentate gyrus structure and function are more vulnerable than the other hippocampal subfields in MS, we hypothesized that pattern separation, a task that has not been tested in MS before, would be impaired very early during the course of the disease, potentially explaining their early deficit in encoding.

## 2 METHODS

### 2.1 Participants

Nineteen patients with early multiple sclerosis (PweMS) were prospectively recruited at Bordeaux University Hospital within 6‐to‐18 months after a first neurological episode suggestive of MS. According to these criteria, patients included in this study were either patients with clinically isolated syndrome highly suggestive of MS with at least two asymptomatic cerebral lesions on Fast Fluid‐Attenuated Inversion‐Recovery (FLAIR) images, or patients with relapsing‐remitting MS according to revised McDonald's criteria (Polman et al., 2005). Exclusion criteria were age under 18 or over 60 years, previous history of other neurological or psychiatric disorder, inability to perform computerized tasks, decreased visual acuity or diplopia, and corticosteroid pulse therapy within 1 month preceding testing. Nineteen healthy control (HC) subjects were tightly matched for age, gender and educational level to PweMS. They were free of neurologic, psychiatric, or systemic diseases, and drug or alcohol abuse. This work was an ancillary study of the SCICOG protocol (NCT01865357), which was approved by the local ethics committee. Written informed consent was obtained prior to participation.

### 2.2 Neuropsychological assessment

**Figure 1:**
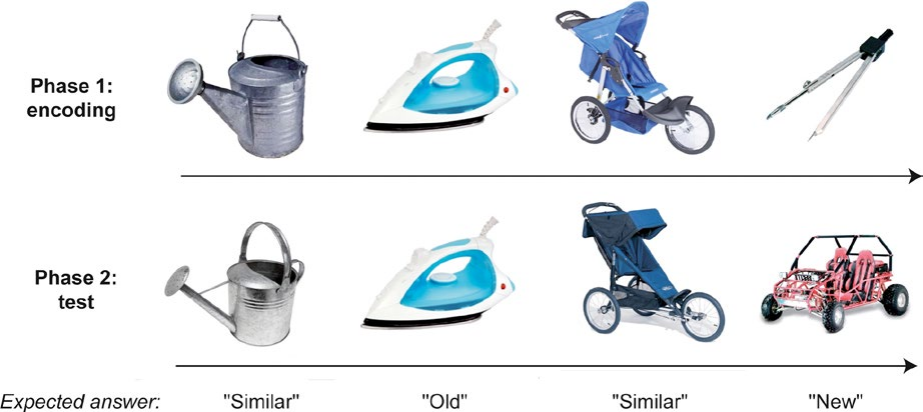
Design of the Mnemonic Similarity Test (MST) and examples of pictures. During the first phase, participants encode a series of pictures. The second phase is a surprise recognition memory test in which participants have to respond “old”, “new” or “similar” to a series of pictures that are exactly the same old images from study, novel foils, or lures that are related but not identical to the studied items. Pattern separation score is the rate of “similar” responses given to the lure minus the rate of “similar” responses given to the foils. (Pictures are those from the MST, freely available at http://faculty.sites.uci.edu/starklab)

All subjects performed the Computerized Speed Cognitive Test (CSCT, a digit/symbol substitution test) (Ruet, Deloire, Charré‐Morin, Hamel, & Brochet, 2013) to assess information processing speed. In order to assess “global” visuospatial episodic memory, all subjects performed the Brief Visuospatial Memory Test revised (BVMT‐R) (Strober et al., 2009). Each participant also completed a standard questionnaire concerning depressive symptoms (Beck Depression Inventory, BDI). Pattern separation and completion were tested in patients and healthy controls with the Mnemonic Similarity Test (MST), also known as the Behavioral Pattern Separation Task (BPS‐O for Mac OS X, version 0.80, http://faculty.sites.uci.edu/starklab/mnemonic-similarity-task-mst) (Kirwan & Stark, 2007). This computerized test consists of two phases. The first phase is an incidental encoding phase where participants are exposed to 128 pictures shown for 2 s with an interstimulus interval of 0.5 s. During this phase, participants have to identify “indoor” and “outdoor” objects via a button press, in order to maintain their attention. Immediately after this encoding phase (~2 min later), the second phase is an unexpected test phase where participants are exposed to 192 pictures with similar exposition time (2 s). One‐third of pictures are exact repetitions of pictures shown in phase 1 (“targets”), one‐third of pictures are completely new objects (“foils”) and one‐third of pictures represent similar but slightly different objects (“lures”). Participants are asked to identify “old”, “new” or “similar” pictures via a button press (Figure 1). Pattern separation score was calculated as the rate of “similar” responses given to the “lure” items minus the rate of “similar” responses given to the “foils” (to correct for response bias). Pattern completion score was the rate of “old” responses given to the “lure” items minus the rate of “old” responses given to the “foils”.

### 2.3 Statistical analyses

Statistical analyses were performed with Prism software 6 (Graphpad). Nominal variables were tested with Chi‐square test. The distribution of all continuous data was tested with the Shapiro–Wilk normality test. Then, Student's *t*‐test after ensuring that variances were equal or Mann–Whitney test was used as appropriate, to compare the two groups. Considering the issue of multiple comparisons, Holm–Bonferroni corrections was used when appropriate, to reduce the risk of type I error (Holm, 1979). Cohen's d was used to measure the effect size. Relationships between quantitative variables were assessed using correlation coefficients (Pearson or Spearman according to statistical distribution). All tests were two‐sided, with a threshold for type I error set at α=0.05.

## 3 RESULTS

### 3.1 Demographical and clinical characteristics of patients and controls

No statistically significant difference between groups was found for age, gender and education level. According to our inclusion criteria, 12 persons were diagnosed with clinically isolated syndrome (CIS) at the time of testing and seven persons had converted to clinically definite multiple sclerosis, less than 18 months after a first clinical relapse. As expected, physical disability was very low but PweMS showed higher depression scores in the BDI than healthy controls, as usually described (Chiaravalloti & DeLuca, 2008) (Table 1).

**Table 1:** Demographical and clinical data of patients and healthy controls. Patients and controls were prospectively included and matched for age, gender and educational level

	Early MS	Healthy controls	*p*‐value
Mean age, years (*SD*)	37.11 (10.24)	34.32 (11.07)	.43
Sex ratio (F/M)	16/3	15/4	.68
Mean education level, years (*SD*)	13.74 (2.10)	14.42 (2.14)	.33
Mean disease duration, months (*SD*)	11.68 (5.84)	–	–
Median EDSS score [range]	1.5 [0 – 3.5]	–	–
Median depression (BDI) score [range]	6 [1–34]	1 [0–11]	.0021

BDI, Beck Depression Inventory; EDSS, Expanded Disability Status Scale; MS, Multiple Sclerosis; *SD*, Standard Deviation.

### 3.2 Neuropsychological testing and mnemonic similarity task

**Figure 2:**
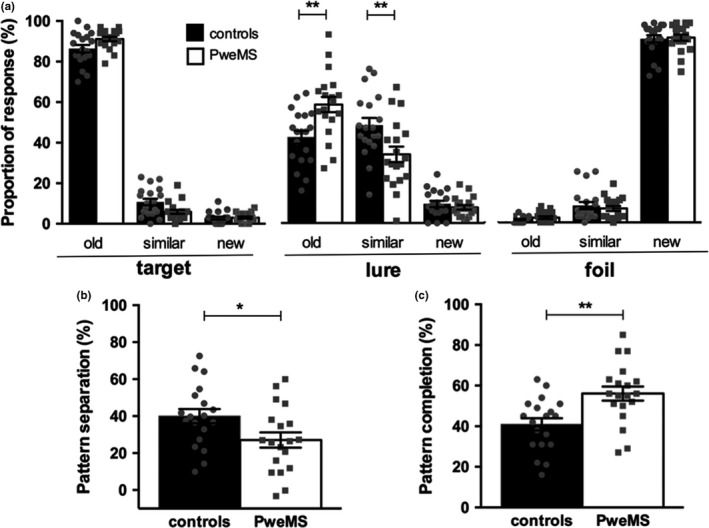
Results of the Mnemonic Similarity Test (MST) in patients with early MS (PweMS) and healthy controls. a: Response accuracy during the test phase for each type of pictures/stimuli (“target”, “new” or “foil”). b and c: Pattern separation and pattern completion scores. Each dot represents a participant. Results are presented as mean ± *SEM*. *p < .05, **p < .01

The neuropsychological testing showed no difference among groups for information processing speed performance. There was also no difference for “global” visuospatial episodic memory tests as assessed with the BVMT‐R regarding learning, long‐term recall or recognition (Table 2). However, we found a significant decrease in pattern separation performance in PweMS compared to healthy controls matched on age, gender and education level (27.07 vs. 40.01, *p* = .030 after Holm‐Bonferroni correction) with a large effect size (Cohen's *d* = 1.02). We also found a significantly higher pattern completion rate in PweMS than in healthy controls (56.11 vs. 40.95, *p* = .004 after Holm‐Bonferroni correction, *d* = 1.07). Decreased pattern separation and increased pattern completion scores in PweMS compared to healthy controls were explained by lower accuracy in identifying the “lure” items as “similar” (33.84 vs. 48.26, *p* = .0096, *d* = 3.86) and, as a consequence, by a higher identification of “old” items as “similar” (58.47 vs. 42.32, *p* = .0022, *d* = 4.65) (Figure 2).

**Table 2:** Neuropsychological results of patients and healthy controls

	Early MS	Healthy controls	*p*‐value
Information processing speed			
CSCT, mean (*SD*)	52.79 (7.87)	52.21 (6.73)	.81
Visuospatial episodic memory			
BVMT‐R learning, mean (*SD*)	29.53 (3.20)	28.68 (6.07)	.60
BVMT‐R delay recall, median [range]	11 [10–12]	12 [7–12]	.23
BVMT‐R recognition, median [range]	6 [5–6]	6 [5–6]	.61

BVMT‐R, Brief visuospatial memory test revised; CSCT, Computerized Speed Cognitive Test; MS, Multiple Sclerosis; *SD*, Standard Deviation.

No correlation was found between pattern separation rate and disability (EDSS) or depression (BDI) in PweMS (respectively *r* = −.17, *p* = .49 and *r* = −.076, *p* = .75).

## 4 DISCUSSION

In this study, we showed decreased pattern separation performance and higher pattern completion performance in a sample of PweMS characterized by the absence of detectable information processing speed and visuospatial memory impairment which suggest early dysfunction of the dentate gyrus in MS.

While verbal or visuospatial episodic memory impairment have been reported from the stage of CIS in previous studies (Feuillet et al., 2007; Planche et al., 2016), we did not detect any difference between groups in BVMT‐R subscores in this sample. This might be due to the fact that our subjects had a rather high educational level, meaning a high cognitive reserve that can protect them against memory impairment as measured with conventional tests (Sumowski et al., 2014). Furthermore, the small sample size with nonseverely affected PweMS might lack statistical power to capture memory decline with the BVMT‐R. Interestingly enough, this sample of patients offers the opportunity to dissect the earliest events that may precede memory decline and highlights that behavioural pattern separation tasks can identify subtle memory decline in patients deemed “cognitively intact” on conventional neuropsychological testing (Feinstein, Lapshin, O'Connor, & Lanctôt, 2013).

This interpretation is controlled for confounders and sources of variability. First, patients and healthy controls were tightly matched on age because it has been demonstrated that pattern separation performance is negatively correlated with age (Holden, Toner, Pirogovsky, Kirwan, & Gilbert, 2013; Stark, Yassa, Lacy, & Stark, 2013). Second, patients and controls performed equally in the CSCT, excluding encoding deficit due to impaired attention or slowing of information processing speed. Third, even though PweMS were more depressed than healthy controls, BDI score did not correlate with pattern separation scores, potentially excluding bias linked to psychiatric comorbidity, poor motivation or attention deficit. Finally, because patients with decreased visual acuity or diplopia due to MS relapse were not included in this study, we assume that our results were not due to visual processing impairment in PweMS.

Loss of pattern separation performance has already been described in neurodegenerative disorders such as Alzheimer's disease (Ally, Hussey, Ko, & Molitor, 2013; Stark et al., 2013) and neurodevelopmental disorders such as schizophrenia (Das, Ivleva, Wagner, Stark, & Tamminga, 2014). We extend for the first time this concept to a neuro‐immune disorder such as MS. Interestingly, during the course of Alzheimer's disease, deficit in pattern separation occurred prior to dementia, from the stage of mild cognitive impairment (MCI) (Stark et al., 2013). We also demonstrated here that loss of pattern separation performance occurred early during the course of MS, even in apparently “cognitively intact” patients as measured with CSCT or BVMT‐R. Then, the behavioral pattern separation task provides a sensitive measure of episodic memory impairment and may be useful to screen for early hippocampal dysfunction during the course of MS.

Studies on rodents have demonstrated that some hippocampal subfields can be more vulnerable than others to peripheral inflammation, cytokines release or microglial activation (Czerniawski & Guzowski, 2014) and our group has pinpointed the early vulnerability of the dentate gyrus at the early phase of experimental MS (Planche et al., 2017). Given that dentate gyrus morphological abnormalities have been described in MS (Gold et al., 2010; Rocca et al., 2015), and given the particular importance of the dentate gyrus for pattern separation, our results provide additional neuropsychological support for an early dentate gyrus disruption during the course of MS. This selective vulnerability of dentate granular neurons at the early stage of MS could be related to a differential pattern of microglial activation in the dentate gyrus compared to other hippocampal subfields (Di Filippo et al., 2016; Planche et al., 2017), but the exact underlying molecular mechanisms remain unclear.

The bias towards pattern completion (i.e., incorrectly recognized a “similar” item as an “old” studied item) measured in PweMS appeared to be the counterpart of pattern separation impairment because these two processes work concurrently (Ally et al., 2013). Indeed, previous rodents and human studies have suggested that when dentate gyrus is unable to perform pattern separation, CA3 (and its connections to CA1) could balance this deficit by overwriting previous representations (Neunuebel & Knierim, 2014; Yassa & Stark, 2011).

During encoding, pattern separation minimizes overlaps between similar incoming activity patterns. Then, impairment in pattern separation should lead to non‐efficient encoding abilities and our findings are consistent with previous work showing that encoding is impaired early in MS whereas storage, retrieval, and recognition are relatively preserved (DeLuca et al., 1998; Müller et al., 2013; Olivares et al., 2005). However, and contrary to previous conclusions, we argue that encoding impairment in early MS would not be secondary to attentional deficit or dysexecutive syndrome due to global brain damage or demyelination but to more specific hippocampal damage resulting in loss of pattern separation performance. It supports early dentate gyrus disruption because lesions in the dentate gyrus inputs are known to impair encoding but not retrieval (Yassa & Stark, 2011).

The main limitation of this work is its small sample size and this pilot work needs to be confirmed in larger studies. Future MRI experiments will need to parallel the specific impairment of pattern separation that we highlighted here with dentate gyrus preferential dysfunction/degeneration. It will be also interesting to look at functional and structural hippocampal connectivity in PweMS with pattern separation impairment, to assess the integrity of the fornix, prefrontal cortex, entorhinal cortex and perforant pathway, that can be potentially damaged in MS and involved in pattern separation (Bennett & Stark, 2016; Pidgeon & Morcom, 2016; Rocca et al., 2015). Furthermore, longitudinal studies will be needed in order to demonstrate that pattern separation impairment precedes and predicts larger deficit in episodic memory, as measured with conventional tests.

## 5 CONCLUSION

By demonstrating selective loss of pattern separation performance in PweMS, we have potentially identified a more sensitive test to detect early cognitive impairment in MS to better screen patients eligible for neuroprotective clinical trials or neurorehabilitation strategies. Furthermore, these data support theories arguing that acute inflammation does not affect the hippocampus homogeneously but specifically disrupts computation tasks supposed to be particularly dependent on the integrity of the dentate gyrus (Czerniawski & Guzowski, 2014; Planche et al., 2017). It therefore provides functional evidence to extend to the human disease the concept of the selective dentate gyrus disruption observed at the early phase of experimental MS in rodents. However, this hypothesis still needs to be confirmed with morphological and functional MRI studies in patients with early MS.

## DISCLOSURES

VP received travel expenses and/or consulting fees from ARSEP Foundation, Biogen, Teva‐Lundbeck and Merk‐Serono unrelated to the submitted work. AR received research grants and/or consulting fees from Novartis, Biogen, Merck‐Serono, Bayer Healthcare, Roche, Teva, and Genzyme unrelated to the submitted work. BB serves on scientific advisory boards for or has received honoraria or research support for its institution from Biogen‐idec, Merck‐Serono, Novartis, Genzyme, Teva, Roche, Medday and Bayer outside the submitted work.
